# Nanoparticles with Antioxidant Activity

**DOI:** 10.3390/antiox14020221

**Published:** 2025-02-15

**Authors:** Regina G. Daré, Sueli O. S. Lautenschlager

**Affiliations:** 1 Institute of Biomedical Sciences, University of São Paulo, 1524 Professor Lineu Prestes Avenue, São Paulo 05508-000, SP, Brazil; 2 Department of Basic Health Sciences, State University of Maringá (UEM), Maringá 87020900, PR, Brazil; 3 Post-Graduate Program in Pharmaceutical Sciences, State University of Maringá (UEM), Maringá 87020900, PR, Brazil

Oxidative stress is commonly defined as an imbalance between reactive oxygen species (ROS) production and an organism’s ability to neutralize them via antioxidant defense mechanisms, leading to damage to biomolecules, including lipids, proteins, and DNA [1,2]. Harman (1956) [3] introduced the theory that linked free radicals to aging, suggesting that oxidative damage accumulates over time, while Sies (1985) [4] later refined this concept, defining oxidative stress as a shift in the pro-oxidant/antioxidant balance in favor of the former, resulting in cellular damage. Although low to moderate levels of ROS are essential for physiological processes such as cellular proliferation, differentiation, and migration, excessive ROS levels contribute to the development of chronic diseases, neurodegenerative disorders, cardiovascular conditions, and aging [5,6]. To counteract oxidative stress, antioxidants are critical. These molecules inhibit the oxidation of other molecules by neutralizing ROS and other free radicals, thus preventing oxidative damage to cells and tissues [7].

Recent advancements in nanotechnology have led to the introduction of nanoparticles as innovative antioxidant carriers in nanomedicine. Additionally, some types of nanoparticles, particularly inorganic ones, exhibit intrinsic antioxidant activity by directly reacting with ROS or mimicking natural antioxidant enzymes [8]. Whether through acting as antioxidant carriers or exerting their intrinsic antioxidant abilities, nanoparticles offer several advantages over conventional pharmaceutical systems. These include enhanced stability, bioavailability, and targeted delivery. For instance, nanoparticles can protect antioxidants from degradation, as many are susceptible to oxidation or degradation under physiological conditions [9]. Drug encapsulation within nanoparticles can also improve the solubility of poorly water-soluble antioxidants, thereby enhancing their bioavailability [10]. Moreover, nanoparticles enable the controlled and sustained release of antioxidants, prolonging their therapeutic effects and minimizing their dose frequency [8]. Targeted delivery systems, such as ligand-functionalized nanoparticles, further enhance these benefits by directing antioxidants specifically to tissues or cells affected by oxidative stress, improving their therapeutic efficacy and reducing any potential side effects [11]. These properties make nanoparticles highly effective for therapeutic applications that require efficient antioxidant delivery.

This Special Issue, “Nanoparticles with Antioxidant Activity”, comprises several studies focused on the synthesis, characterization, and therapeutic potential of various nanoparticles aimed at mitigating oxidative stress in biological systems. Herein, we will summarize the key contributions of these studies.

Nanoparticles have the ability to enhance the bioavailability and therapeutic efficacy of both natural and synthetic antioxidants, particularly for compounds with poor solubility. By employing strategies such as covalently linking antioxidants to nanoparticles, entrapping them in nanogels or hollow particles, or encapsulating them within nanoparticles of various origins, their stability and solubility can be significantly improved. This approach not only protects antioxidants from degradation but also improves their interactions with ROS, ultimately increasing their effectiveness in reducing oxidative stress in biological systems [11]. For example, in this Special Issue, Abdol Wahab et al. [12] reviewed nanocarriers for the oral delivery of astaxanthin, an antioxidant with poor oral bioavailability due to its lipophilicity. The authors highlighted several nanocarrier systems, such as nanoemulsions, solid lipid nanoparticles (SLNs), chitosan-based nanoparticles, and PLGA-based nanoparticles, which have shown great promise in overcoming the limitations of astaxanthin’s bioavailability. The study demonstrated that nanoemulsions enhanced astaxanthin’s permeability by up to 80% in simulated intestinal conditions. Furthermore, chitosan-based nanoparticles exhibited improved dispersibility in the gastrointestinal environment and prolonged astaxanthin release, ensuring more consistent antioxidant activity. Similarly, SLNs were shown to protect astaxanthin from degradation in simulated gastrointestinal fluids, significantly enhancing its stability and antioxidant efficacy. These findings emphasize the potential of nanocarriers in optimizing the therapeutic use of astaxanthin and similar antioxidants.

As previously mentioned, inorganic nanoparticles have gained attention due to their inherent antioxidant properties. Metal nanoparticles such as those containing platinum (Pt) [13] or nickel oxide (NiO) [14] have been studied for their antioxidant properties. However, the effectiveness of these nanoparticles as antioxidants depends on factors such as their chemical composition, surface charge, particle size, surface-to-volume ratio, and surface coating [15]. This Special Issue brings together research articles focusing on the inorganic nanomaterials Prussian blue nanoparticles, gold nanoparticles, and cerium oxide nanoparticles. Prussian blue nanoparticles exhibit redox capabilities and have been studied for applications in medicine, particularly in detoxification and photothermal therapies [16]. Gold nanoparticles, with their unique surface plasmon resonance, offer versatile applications ranging from drug delivery to diagnostic imaging, and have also demonstrated antioxidant and anti-inflammatory effects [17,18]. Meanwhile, cerium oxide nanoparticles (nanoceria) exhibit redox properties, allowing them to act as antioxidants. They can switch between the Ce^3+^ and Ce^4+^ oxidation states, making them effective in scavenging ROS and protecting against oxidative stress [19].

One of the advantages of encapsulating antioxidants into nanoparticles is the ability to control the release of the therapeutic agent at the target tissue [20]. This controlled release enhances the stability of antioxidants, protecting them from premature degradation and ensuring a more efficient and localized therapeutic effect. Nanoparticles can be engineered to respond to specific stimuli such as pH variations, temperature, or enzymatic activity within the target tissue, allowing for a highly targeted and responsive delivery system [20,21]. Such stimuli-responsive nanoparticles ensure that antioxidants are released only when and where needed, further optimizing therapeutic outcomes. This targeted approach improves antioxidants’ bioavailability and efficacy, significantly reducing potential side effects that may arise from systemic exposure or off-target effects. Additionally, encapsulating antioxidants within nanoparticles can enhance their interaction with ROS, allowing for more efficient oxidative stress neutralization at the cellular level [13,22].

Nanoparticles with antioxidant activity have been widely reported to treat oxidative stress-related pathologies. Herein, we collected articles that demonstrate the effectiveness of nanoparticles in treating or preventing some of these conditions. For instance, nanoceria has shown protective effects against mitochondrial dysfunction and cardiac hypertrophy by scavenging ROS and enhancing mitochondrial biogenesis [23]. Specifically, nanoceria was found to reduce intracellular ROS levels, upregulate the antioxidant enzymes superoxide dismutase and catalase, and enhance the expression levels of genes involved in mitochondrial biogenesis (e.g., PGC-1α) and fusion (e.g., MFN2). These effects led to improved mitochondrial function, making nanoceria a promising candidate for treating oxidative stress-induced cardiovascular conditions. Additionally, curcumin-loaded nanostructured lipid carriers (nano-curcumin) have shown efficacy in mitigating neurotoxicity caused by cypermethrin-induced oxidative stress, inflammation, and apoptosis in rat brains [24]. In this study, nano-curcumin not only improved the activity of antioxidant enzymes like catalase and superoxide dismutase but also reduced lipid peroxidation and pro-inflammatory cytokine levels such as IL-6, IL-1β, and TNF-α. Furthermore, nano-curcumin was able to suppress the activation of apoptotic markers, including caspase-3 and caspase-9, thus providing significant neuroprotective effects. This highlights the potential of nanoparticle-based delivery systems in enhancing the bioavailability and efficacy of antioxidants in treating neurodegenerative disorders.

Despite their therapeutic promise, nanoparticles pose potential risks. Nanoparticle toxicity can arise from their unique physicochemical properties, such as their size, shape, surface charge, and chemical composition [25], as well as from their interactions with biological systems, including protein corona formation [26]. These factors influence their cellular uptake, biocompatibility, and mechanisms of toxicity, such as oxidative stress, inflammation, and genotoxicity [25,27]. Metal-based nanoparticles (e.g., iron, copper, and zinc oxides) and carbon nanotubes are particularly prone to eliciting oxidative stress [27]. Therefore, characterizing nanoparticle properties and evaluating their nano-toxicity is imperative for balancing their benefits and potential adverse effects.

This Special Issue presents seven pieces of original research and two reviews that discuss various aspects of nanoparticle-based antioxidant therapies and their biomedical applications. The articles provide significant data regarding the physicochemical characterization of nanoparticles, their mechanisms of antioxidant delivery, and pre-clinical therapeutic efficacy. These contributions will serve as a foundation for future research aimed at optimizing nanoparticle-based interventions for oxidative stress-induced pathologies. While the therapeutic potential of these nanoparticles is evident, further studies are needed to optimize their biocompatibility, stability, and delivery efficiency. Most existing studies are based on preclinical research (in vitro and in vivo), leaving significant gaps in our understanding of their full toxicity profile and therapeutic efficacy in clinical settings. To advance the field, comprehensive clinical investigations are crucial.
